# Endemic and Emerging Arboviruses in Domestic Ruminants in East Asia

**DOI:** 10.3389/fvets.2020.00168

**Published:** 2020-04-07

**Authors:** Tohru Yanase, Katsunori Murota, Yoko Hayama

**Affiliations:** ^1^Kyushu Research Station, National Institute of Animal Health, NARO, Kagoshima, Japan; ^2^Viral Disease and Epidemiology Research Division, National Institute of Animal Health, NARO, Tsukuba, Japan

**Keywords:** arthropod-borne virus, cattle, *Culicoides*, mosquito, orbivirus, orthobunyavirus, rhabdovirus

## Abstract

Epizootic congenital abnormalities caused by Akabane, Aino, and Chuzan viruses have damaged the reproduction of domestic ruminants in East Asia for many years. In the past, large outbreaks of febrile illness related to bovine ephemeral fever and Ibaraki viruses severely affected the cattle industry in that region. In recent years, vaccines against these viruses have reduced the occurrence of diseases, although the viruses are still circulating and have occasionally caused sporadic and small-scaled epidemics. Over a long-term monitoring period, many arboviruses other than the above-mentioned viruses have been isolated from cattle and *Culicoides* biting midges in Japan. Several novel arboviruses that may infect ruminants (e.g., mosquito- and tick-borne arboviruses) were recently reported in mainland China based on extensive surveillance. It is noteworthy that some are suspected of being associated with cattle diseases. Malformed calves exposed to an intrauterine infection with orthobunyaviruses (e.g., Peaton and Shamonda viruses) have been observed. Epizootic hemorrhagic disease virus serotype 6 caused a sudden outbreak of hemorrhagic disease in cattle in Japan. Unfortunately, the pathogenicity of many other viruses in ruminants has been uncertain, although these viruses potentially affect livestock production. As global transportation grows, the risk of an accidental incursion of arboviruses is likely to increase in previously non-endemic areas. Global warming will also certainly affect the distribution and active period of vectors, and thus the range of virus spreads will expand to higher-latitude regions. To prevent anticipated damages to the livestock industry, the monitoring system for arboviral circulation and incursion should be strengthened; moreover, the sharing of information and preventive strategies will be essential in East Asia.

## Introduction

Arthropod-borne (arbo-) viruses are transmitted by hematophagous arthropods. The arboviruses include various taxonomic groups, such as *Rhabdoviridae, Nairoviridae, Peribunyaviridae, Phenuiviridae, Orthomyxoviridae, Flaviviridae, Togaviridae, Reoviridae*, and *Asfarviridae* (1, 2). They cause a broad spectrum of illness ranging from asymptomatic to severe and fatal disease in vertebrate hosts. Because some of the arboviruses have affected human and domestic animal health, the efforts to prevent and control the diseases caused by arboviruses continue, but novel arboviruses have continuously emerged and have sometimes been involved in human and animal diseases. The battle thus seems to have no end.

Bluetongue virus (BTV; the genus *Orbivirus*, the family *Reoviridae*) is probably the most well-known arbovirus affecting livestock ruminant productions in the world (3). The virus causes severe illness in ruminants and its presence can act as a barrier for international trades of animals and animal products. Since end of the last century, the global range of BTV has remarkably changed (4). The incursion of BTV serotype 8 into northern Europe caused large economic damage to the livestock industry. Another important arbovirus, epizootic hemorrhagic disease virus (EHDV; the genus *Orbivirus*), also has been regarded as a threat to European counties in recent years, because its outbreaks has been reported in the countries bordering the region (3, 5). The ruminant diseases caused by both viruses have been listed as notifiable terrestrial animal diseases by World Organization for Animal Health (OIE) and well-investigated through the world. In addition, recent appearance of an emerging arbovirus, Schmallenberg virus (SBV), in northern Europe (6) has caught the world's attention (7). However, many other arboviruses, which are not listed by OIE, also have been involved in ruminant illness and their epizootic might have severely impacted on the livestock industry in each region. Unfortunately, the situation of arbovirus infections of ruminant livestock remains uncertain in many parts of the world.

East Asia, i.e., the eastern subregion of Asia, has the largest human population in the world, and the livestock industry is an important component of the lives of the region's populations. The estimated global distribution data for livestock animals indicated that East Asia is one of the regions with the highest population density of domestic ruminants in the world (8). Significant concern about the health and safety of livestock for consumption has thus been expressed. Unfortunately, along with other important diseases, a variety of arbovirus infections has probably damaged the production of domestic ruminants (Tables 1, 2), although there is insufficient statistical data at this time to draw any conclusion.

**Table 1:** Arboviruses associated with domestic ruminants in East Asia.

**Family**	**Genus**	**Species**	**Virus/serotype (abbreviation)**	**Arthropod vectors**	Geographical location of isolation/detection^*^
*Peribunyaviridae*	*Orthobunyavirus*	*Akabane orthobunyavirus*	Akabane virus (AKAV)	*Culicoides* spp.	**CHN**, **JPN**, **ROK**, **TWN**
		*Aino orthobunyavirus*	Aino virus (AINOV)	*Culicoides* spp.	*CHN*, **JPN**, **ROK**, TWN
		*Peaton orthobunyavirus*	Peaton virus (PEAV)	*Culicoides* spp.	*CHN*, **JPN**, *ROK*, TWN
		*Schmallenberg orthobunyavirus*	Sathuperi virus (SATV)	*Culicoides* spp.	**JPN**, *ROK*
			Shamonda virus (SHAV)	*Culicoides* spp.	**JPN**, *ROK*
		*Batai orthobunyavirus*	Batai virus (BATV)	Mosquitoes	**CHN**, **JPN**
*Phenuiviridae*	*Banyangvirus*	*Huaiyangshan banyangvirus*	Severe fever with thrombocytopenia syndrome virus (SFTSV)	Ticks	**CHN**, **JPN**, **ROK**
*Nairoviridae*	*Orthonairovirus*	*Crimean-Congo hemorrhagic fever orthonairovirus*	Crimean-Congo hemorrhagic fever virus (CCHFV)	Ticks	**CHN**
*Reoviridae*	*Orbivirus*	*Epizootic haemorhagic virus*	Epizootic hemorrhagic disease virus 1 (EHDV-1)	*Culicoides* spp.	**JPN**
			Epizootic hemorrhagic disease virus 2 (EHDV-2)	*Culicoides* spp.	**JPN**, **ROK**, **TWN**
			Epizootic hemorrhagic disease virus 6 (EHDV-6)	*Culicoides* spp.	*JPN*
			Epizootic hemorrhagic disease virus 7 (EHDV-7)	*Culicoides* spp.	**CHN, JPN**
			Epizootic hemorrhagic disease virus 10 (EHDV-10)	*Culicoides* spp.	**JPN**
		*Bluetongue virus*	Bluetongue virus 1 (BTV-1)	*Culicoides* spp.	**CHN**, *JPN*, **ROK**
			Bluetongue virus 2 (BTV-2)	*Culicoides* spp.	**CHN**, **JPN**, *ROK*, **TWN**
			Bluetongue virus 3 (BTV-3)	*Culicoides* spp.	**CHN**, **JPN**, **ROK**
			Bluetongue virus 4 (BTV-4)	*Culicoides* spp.	**CHN**, *ROK*
			Bluetongue virus 5 (BTV-5)	*Culicoides* spp.	**CHN**
			Bluetongue virus 7 (BTV-7)	*Culicoides* spp.	**CHN**, *ROK*
			Bluetongue virus 9 (BTV-9)	*Culicoides* spp.	**CHN, JPN**
			Bluetongue virus 12 (BTV-12)	*Culicoides* spp.	**CHN**, **JPN**, **TWN**
			Bluetongue virus 15 (BTV-15)	*Culicoides* spp.	**CHN**, *ROK*
			Bluetongue virus 16 (BTV-16)	*Culicoides* spp.	**CHN**, **JPN**, *ROK*
			Bluetongue virus 20 (BTV-20)	*Culicoides* spp.	*JPN*
			Bluetongue virus 21 (BTV-21)	*Culicoides* spp.	**CHN**, **JPN**
			Bluetongue virus 24 (BTV-24)	*Culicoides* spp.	**CHN**
			Unclassified strain XJ1407	*Culicoides* spp.	**CHN**
		*Palyam virus*	Chuzan virus (CHUV)	*Culicoides* spp.	**CHN**, **JPN**, **ROK**, **TWN**
			D'Aguilar virus (DAGV)	*Culicoides* spp.	**JPN**
			Bunyip Creek virus (BCV)	*Culicoides* spp.	**JPN**
		*Yunnan orbivirus*	Yunnan orbivirus (YUOV)	Mosquitoes	**CHN**
		Unclassified	Guangxi orbivirus (GXOV)	NR	**CHN**
		Unclassified	Tibet orbivirus (TIBOV)	Mosquitoes, *Culicoides* spp.	**CHN**
		Unclassified	Sathuvachari virus (SVIV)	Mosquitoes	**JPN**
	*Seadornavirus*	*Banna virus*	Banna virus (BAV)	Mosquitoes	**CHN**
*Rhabdoviridae*	*Ephemerovirus*	*Bovine fever ephemerovirus*	Bovine ephemeral fever virus (BEFV)	Mosquitoes/*Culicoides* spp.	**CHN**, **JPN**, **ROK**, **TWN**
		Unclassified	KSB-1/P/03	NR	**JPN**
	*Ledantevirus*	*Fukuoka ledantevirus*	Fukuoka virus (FUKV)	Mosquitoes/*Culicoides spp*.	**JPN**
*Flaviviridae*	*Flavivirus*	*Japanese encephalitis virus*	Japanese encephalitis virus (JEV)	Mosquitoes	**CHN**, **JPN**, **ROK**, **TWN**
		*West Nile virus*	West Nile virus (WNV)	Mosquitoes	**CHN**, ROK
		*Kyasanur Forest disease virus*	Kyasanur Forest disease virus (KFDV)	Ticks	**CHN**
		*Tick-borne encephalitis virus*	Tick-borne encephalitis virus (TBEV)	Ticks	**CHN**, **JPN**, **ROK**
	Unclassified	Unclassified	Jingmen tick virus (JMTV)	Ticks	**CHN**
*Togaviridae*	*Alphavirus*	*Getah virus*	Getah virus (GETV)	Mosquitoes	**CHN**, **JPN**, **ROK**, **TWN**

*NR, none reported; CHN, mainland China; JPN, Japan; ROK, Republic of Korea; TWN, Taiwan*.

* *Locations with virus isolation (bold letter), serological evidence (Italics), genetic detection (underline) or the latter two (Italics and underline)*.

**Table 2:** Important endemic arbovirus infections in ruminant livestock in East Asia.

**Disease**	**Etiological virus**	**Clinical signs (affected ruminant species)**
Akabane disease	AKAV	Abortion, premature birth, stillbirth, congenital malformations (cattle, buffalo, sheep, goat) Neurological disorders caused by encephalomyelitis (postnatally infected cattle)
Aino virus infection	AINOV	Abortion, premature birth, stillbirth, congenital malformations (cattle, buffalo, sheep, goat)
Chuzan disease	CHUV	Congenital malformaitons (cattle)
Bovine ephemeral fever	BEFV	Sudden fever, anorexia, depression, tachypnea, lacrimation, foamy salivation, arthritis, limping, ananastasia (cattle, buffalo)
Ibaraki disease	EHDV-2 (Ibaraki strain)	Fever, anorexia, lachrymation, salivation, nasal discharge, conjunctival injection, swallowing difficulty (cattle)
Bluetongue	BTV	Fever, nasal discharge, tachpypnea, lethargy, hemorrhages (cattle, sheep, goat) Congenital malformations (cattle)

Akabane disease and bovine ephemeral fever (BEF) are still problematic in East Asia, despite the widespread use of vaccines against them. Outbreaks of other endemic arbovirus infections such as Aino virus infection and Ibaraki disease have occasionally been reported. In addition, recent progress in the research tools for virus hunting have enhanced the detection of the circulation of arboviruses that were previously unrecognized in East Asia (Table 1). Notably, some of the arboviruses are suspected of being associated with ruminant diseases such as epizootic congenital abnormalities, febrile illness, and neurological disorders (Table 3). Many reports about arboviruses infecting domestic ruminants have been provided from mainland China, Japan, the Republic of Korea, and Taiwan, which are part of East Asia. This review provides a compilation of the available knowledge about the arboviruses that are associated with domestic ruminants in the East Asia region at present. Gaps in the research that remain to be addressed and future research directions are also discussed.

**Table 3:** Emerging arboviruses suspected to cause diseases in domestic ruminants in East Asia.

**Virus**	**Clinical signs (affected ruminant species)**
PEAV	Congenital malformations (cattle, experimentally in sheep)
SHAV	Congenital malformations (cattle)
SATV	Congenital malformations (cattle)
EHDV-6	Anorexia, fever, cessation of rumination, salivation, swallowing difficulty (cattle)
EHDV-7	Abortion, stillbirth, salivation, swallowing difficulty (cattle)
DAGV	Congenital malformations (cattle)
YUOV	Neurological disorders (cattle, sheep)

## History of Endemic and Emerging Arboviruses Affecting Domestic Ruminants

### Bovine Ephemeral Fever

BEF is characterized by sudden fever, anorexia, depression, and ananastasia, and it can be regarded as the first recognized arbovirus infection in domestic ruminants in East Asia. Due to the cessation of lactation in dairy cattle and the loss of condition in beef cattle, BEF has had considerable economic impact (9). The etiological virus is bovine ephemeral fever virus (BEFV; species *Bovine fever ephemerovirus*, genus *Ephemerovirus*, family *Rhabdoviridae*). The virus was widely distributed through Africa, the Middle East, Asia, and Australia. Phylogenetic analysis based on the surface glycoprotein (G) gene demonstrated that BEFV isolates are grouped into four lineages with different geographical origins: Africa, the Middle East, Asia, and Australia (9, 10). Recently, several BEFV isolates clustered into Asian lineage have been appeared in Turkey and Iran, indicating its expansion to the westward (11, 12). Outside of East Asia, large BEF epizootics have been reported in Israel, Turkey, and Australia (11, 13, 14).

Old records indicated that epidemics of BEF occurred in the central and western parts of Japan in the late nineteenth century (15). Between 1949 and 1951, ~700,000 cases and 10,000 deaths were reported in the same region (16). The disease was initially called “bovine epizootic fever,” but the etiological virus was later identified as BEFV based on the serological relationship between the Japanese isolate and BEFVs isolated in Australia and South Africa (17). Large- and medium-scale outbreaks of BEF repeatedly occurred in Japan until 1989 (18, 19). Since then, the epidemics had been limited to the southwestern islands of Japan, which are close to Taiwan (20, 21). However, in 2015 a reoccurrence of BEF was reported in the southern end of Kyushu, which is one of the main islands of Japan (22).

From 1955 to 2011, BEF epidemics were often recorded in mainland China (23). China's nationwide sero-surveillance in cattle between 2012 and 2014 demonstrated that the range of BEFV covered most of the area of mainland China (24). Epidemics of BEF have also occurred in Korea, and some of them were probably linked to those in Japan (19). Meteorological analysis demonstrated that a low-jet stream blew from mainland China, along the Korean Peninsula, to western Japan at the peak of BEF epidemic in China and Korea in 1988 and 1991 (19, 25, 26). The stream probably brought the infected vectors and contributed to the BEF expansion to Japan. The seroprevalence of BEFV without clinical cases was demonstrated in Korea between 2009 and 2012 (27). Several intervals of BEF epidemic have been documented in Taiwan between 1967 and 2014 (28).

Although the mortality rate of BEF is known to be <1% (9), a high case-fatality rate due to a novel genogroup of BEFV was observed in several recent outbreaks in mainland China and Taiwan (23, 29). This novel genogroup of BEFV was first isolated in mainland China in 2011 and was then detected in Taiwan and Japan 2 and 4 years later, respectively (22, 23, 30). These reports indicate that the virus rapidly spread in East Asia. The impact of BEF caused by the novel genogroup on dairy and beef production should thus be evaluated.

At laboratory diagnosis, the virus neutralization test (VNT) is generally used to detect rising titers of neutralization antibody in paired acute and convalescent sera from affected cattle which show the typical clinical signs of BEF (14). For serological detection of BEFV, an indirect-ELISA with the recombinant protein of BEFV G gene was also developed (31). In recent years, conventional RT-PCRs targeting the G gene was available to detect BEFV in the diseased cattle (32–34). Real-time RT-PCR and reverse-transcription, loop-mediated isothermal amplification (LAMP) assays were also established for rapid and sensitive detection of BEFV gene (32, 35, 36).

Vaccination is now the current gold standard to prevent BEF. Commercial attenuated and killed vaccines have been developed and widely used in Japan, Korea, China and Taiwan (23, 27, 33, 37–39). Repetitive outbreaks of BEF in East Asia indicate that continuous vaccination program is essential to provide sufficient immunity against BEFV among naïve population and minimize economical loss caused by BEF. Antigenic association between the vaccine strains and recent field isolates of BEF should be assessed to guarantee the vaccine efficacy.

### Ibaraki Disease

During a large epidemic of an acute febrile disease in 1959 and 1960 in Japan, deglutitive difficulty based in the esophageal and laryngopharyngeal musculature was observed among affected cattle (40). A total of 43,793 cases and 4,298 deaths were reported during the epidemic. That febrile disease was at one time called “bluetongue-like disease” because its clinical signs resembled those caused by BTV. The etiological agent was isolated from naturally and experimentally infected cattle and was designated as Ibaraki virus (IBAV), which denotes the location from which it was isolated (40, 41). The clinical signs of IBAV were exhibited by the experimentally infected cattle. IBAV was later identified as a strain of EHDV serotype 2 (EHDV-2) based on a virus neutralization test (42). Smaller outbreaks were reported in southern Japan in 1982 and 1987 (43–45), but no case report was known between 1988 and 2012. A resurgence of IBAV was observed in southern Japan in 2013, although only a few cattle showed the typical symptoms (46).

An occurrence of Ibaraki disease was recognized in Korea in 1982, when the epidemic was observed in southern Japan (47). In Taiwan, IBAV was initially obtained in 1990 from the cerebellum of a calf with non-suppurative encephalitis, and an occurrence of Ibaraki disease was observed at several farms in the same year (48). IBAV was also isolated from the affected cattle and from asymptomatic cattle from the same cowsheds where the affected cattle were raised. The seroprevalence (25–85%) of IBAV in dairy cattle was revealed in Taiwan between 1987 and 1990. There is no available information about Ibaraki disease outside of the above-mentioned regions.

At laboratory diagnosis, detection of neutralization antibody to IBAV and virus isolation from diseased cattle are usually tested (41, 43–46). IBAV-specific RT-PCR based on segment 2 is available to detect the virus from infected cattle (46). A real-time RT-PCR assay for typing of EHDV would be able to detect the presence of IBAV in diagnostic samples (49). Currently, commercial attenuated and killed vaccines to IBAV are prepared in Japan (50, 51).

### Akabane Disease

A large outbreak of abortions, premature births, still births, and congenital malformations in calves was reported in Japan between 1972 and 1975 (52). The number of affected calves reached ~42,000 during the outbreak. The malformed calves had congenital lesions characterized by arthrogryposis-hydranencephaly (A-H) syndrome (53, 54). A serological survey of affected calves demonstrated that Akabane virus (AKAV; species *Akabane orthobunyavirus*, genus *Orthobunyavirus*, family *Peribunyaviridae*) was a causative agent of the disease (54). The virus is widely distributed in Africa, the Middle East, Asia and Australia (55) and severe outbreaks of Akabane disease have sometimes occurred in Japan, Australia, and Israel (52, 56–59).

Although AKAV was first isolated in 1959 from mosquitoes (60), its relationship with ruminant disease had been unknown until the outbreak. The etiology of AKAV in bovine and caprine fetuses was demonstrated by experimental infections of pregnant cows and goats (61, 62). Stillbirths and congenital malformations of neonate lambs caused by AKAV were observed in northern Japan between late 1985 and early 1986 (63). The isolation of AKAV from *Culicoides* biting midges, cattle, and a piglet has also been successful (64–67). In 1984, AKAV was isolated from a postnatally infected calf with encephalitis during a small outbreak in Japan; it was a suspected causative agent (68).

Outbreaks of encephalomyelitis caused by postnatal AKAV infection occurred in 2006 and 2011 with 180 and 165 deaths, respectively (67, 69). The AKAVs isolated in Japan since 1959 are clustered into two genogroups (I and II) (67, 70). Genogroup I AKAVs have frequently been detected and/or isolated from affected cattle with encephalomyelitis (67, 69). These isolates probably have high neurotropic and/or neurovirulence and cause the neurological disorders in cattle. Recent monitoring in Japan revealed that the frequent incursion of genogroup I AKAVs increased the number of postnatal encephalitis cases (65). There have been repeated epidemics of Akabane disease including both *in utero* and postnatal infections in Japan (52, 67).

The first recognition of Akabane disease in Korea was in 1979 (71). Several AKAV isolates were then obtained from aborted fetuses, affected cattle with neurological disorders, and sentinel cattle between 1993 and 2010 (72–75). Serological surveillance of domestic ruminants revealed that frequent incursions of AKAV had occurred, and the virus occasionally spread widely (27, 76–78). Ruminants affected by AKAV have been reported since the first recognition (52, 79). In 2010, a large outbreak of epizootic encephalomyelitis in postnatally infected cattle also occurred in the southern part of Korea (74). The causative AKAV isolates were classified into genogroup I (75). Before that outbreak, several sporadic cases of bovine encephalomyelitis were reported in 2000 and 2001 (80).

In 1992, AKAV was isolated in Taiwan from affected calves with non-suppurative encephalitis and healthy calves (81). The 307 sera collected from 341 affected calves with encephalitis in 1989 and 1990 contained the specific antibodies to AKAV (82). A high seroprevalence (94.3%) of AKAV was also observed in 1994 (82). Since 1998, AKAV has been isolated from mosquitoes, bamboo rats (*Rhizomys pruinosus*), and a goat in the southern part of mainland China (83–87). Large-scale serological surveillance demonstrated that AKAV infection was relatively common in cattle and sheep in most of mainland China (83). However, no detailed description of AKAV outbreaks in mainland China can be obtained from published references, and the impacts of AKAV on the Chinese livestock industries remain unknown.

Diagnosis of Akabane disease is usually conducted by VNT for detection of specific antibodies to AKAV in fetal fluids or precolostrum sera of malformed newborns (55). ELISAs were also established for serological tests to detect antibodies to AKAV (88, 89) and the commercial ELISA kits are often used for the diagnosis and serological surveillance (90–92). The virus may be isolated from aborted fetuses (93). Because the neutralization antibodies to AKAV probably clear the virus and virus infected cells before the delivery of affected neonates, virus isolation is not successful from them. The virus gene is also unable to be detected from most of the affected calves. However, conventional and real-time RT-PCR assays can provide a sensitive detection of the AKAV-specific gene in central nervous system of the postnatally infected cattle with encephalomyelitis (69, 74, 94). Attenuated and inactivated vaccines against AKAV for breeding cows have been developed (95–97) and commercially provided in Japan and Korea.

### Aino Virus Infection

Aino virus (AINOV; species *Aino orthobunyavirus*, genus *Orthobunyavirus*) was first isolated from mosquitoes in Japan in 1964 (98). *Culicoides* biting midges are currently regarded as incriminate vectors for AINOV (65). During the outbreak of bovine congenital abnormalities caused by AKAV in Japan in 1972–1975, the precolostral sera of several affected calves with A-H syndrome contained antibodies to AINOV, but not to AKAV (53). Since then, cases suspected of being associated with AINOV have been sporadically reported in the western part of Japan (99–102). AINOV was also obtained from an aborted bovine fetus with brain lesions (103, 104). These findings indicated that AINOV was involved in abnormal deliveries in the region. Later, teratogenicity characterized by arthrogryposis, hydranencephaly, and cerebellar hypoplasia was demonstrated in neonate calves intrauterinely injected with AINOV (105). Several outbreaks (from 17 to 700 cases) of Aino virus infection in Japan were reported between 1995 and 2006 (52). All known Japanese AINOVs likely belong to a single genogroup, but genetically distant from Australian AINOVs (106–108).

A few cases of Aino virus infection were also reported in Korea, and the virus was isolated from a healthy cow in 1999 (52). The seroprevalence of AINOV was demonstrated in cattle ( ≤ 33.2%) and goats (13.3%) in Korea (27, 77, 78). Although there has been no report of Aino virus infection in China, 10 of 50 AKAV-positive cattle sera collected during the nationwide surveillance of AKAV in mainland China neutralized AINOV (83). A partial genome of AINOV was detected from *Culicoides* biting midges and mosquitoes collected at dairy farms in Taiwan between 2012 and 2015 (109).

In Australia, AINOV was also isolated (110, 111) and its association with congenital abnormalities was reported (112). Shuni virus, which is closely related with AINOV and distributed in Africa and the Middle East, caused abortions and congenital malformations in sheep, goat, and cattle (113), and neurological disorders in cattle (114).

VNT for precolostral sera of the affected neonates is the most common diagnostic test at present. Commercial inactivated vaccines for breeding cattle are available in Japan and Korea (51, 115).

### Chuzan Disease

From the winter of 1985 to the spring of 1986, an epizootic of congenital abnormalities of calves with hydranencephaly-cerebellar hypoplasia (HCH) syndrome occurred in southern Japan (116). Unlike Akabane disease, the affected calves did not show arthrogryposis. Approximately 2,400 affected calves were reported during the epizootic. In the autumn of 1985, a virus classified as a member of the *Orbivirus* genus was isolated from *Culicoides* biting midges and bovine blood samples collected in the epizootic region; it was tentatively designated as Chuzan virus (CHUV) (117). The etiological role of CHUV in the epizootic was suggested by serological tests for the affected calves (116). In addition, a newborn calf with HCH syndrome was delivered from a dam experimentally infected with CHUV, and the disease caused by CHUV was named Chuzan disease (118). It was later reported that CHUV is serologically cross-reacted with Kasba virus (KASV), which was isolated in India in 1957 and is a serotype of the species *Palyam virus* (119). Recent genetical characterization of Palyam virus group also demonstrated the closed relationship between CHUV, KASV, and Vellore virus, which was isolated in India in 1956 (120, 121). After the large 1985–1986 outbreak in southern Japan, the disease has sporadically occurred there.

The isolation of the virus and its seroprevalence (91%) among dairy cattle in Taiwan in 1993 were reported (122). In Korea, the prevalence of CHUV has been recognized since 1993, but only a few sporadic cases of affected calves have been reported in recent years (27, 52, 77). Two isolates of CHUV were obtained from sentinel cattle in the southern part of mainland China in 2012 (123, 124). CHUV was also detected from yaks in the Qinghai-Tibetan Plateau in 2016–2017, although no affected case was reported (125). The impact of CHUV in mainland China remains unclear.

Presence of antibody to CHUV in precolostral serum from abnormal calves indicates its association with lesions in central nervous system (126). For serological diagnosis, VNT is appropriate. Inactivated vaccines against CHUV for breeding cows was developed and are commercially available in Japan and Korea (51, 115, 127).

### Bluetongue

Bluetongue is an infectious hemorrhagic disease in domestic ruminants and is caused by BTV, which belongs to the genus *Orbivirus* and consists of at least 27 serotypes (128). *Culicoides* biting midges play a principal role in its transmission. The virus is widely distributed through the world. The circulation of a variety of BTV serotypes has been detected in East Asia. In 1974, bovine sera shown to be BTV-positive by a complement-fixation (CF) test were obtained from cattle in southern Japan (129). Thereafter, at least eight BTV serotypes have been found in Japan based on virus isolation and serological surveillance (130–132). In 1994 and 2001, a total of 23 and 45 sheep, respectively, on the same farm in central Japan developed typical symptoms of bluetongue (131). Twenty-three cattle also developed bluetongue in the same region in 1994.

Suspected bluetongue cases in sheep were first reported in the southern part of mainland China in 1974, and the virus was isolated in 1979 during an outbreak of bluetongue-like disease (133, 134). The prevalence of 12 BTV serotypes in mainland China was determined based on virus isolation (135). A probable novel serotype of BTV spread in the northwestern part of China in 2014 (136). Extensive research including risk analyses of BTV spread were conducted in areas with large sheep populations (137–139).

The first BTV isolation in Taiwan was in 2003 (140). The circulation of two BTV serotypes in Taiwan has been identified to date (141). A retrospective study revealed that antibodies to BTV have been present in goat sera in Taiwan since 1989. However, no clinical cases of bluetongue have been observed. In Korea, BTV-1 was isolated from a bovine blood sample collected at an abattoir in 2012 (142), and thereafter, the circulation of BTV was confirmed in goats, deer, and dairy cattle by serological surveillance (143–145). Seven BTV serotypes have been detected in Korea to date. Recently, BTV-3 was also obtained from a blood sample of cattle reared in Korea (146).

The genomic analysis of BTV isolates in East Asia demonstrated that most of them cluster within the eastern BTV topotype from Asia and Australia (132, 135, 146–150). However, a part of segments of Chinese BTV-7 isolate probably originated from those of African BTV isolates which cluster within the western BTV topotype (151). The finding suggests that genetic reassortment events between different topotypes have occurred in regions neighboring to East Asia.

Various serological and molecular assays have been developed and used for laboratory diagnosis (152). Competitive ELISA, VNT, and conventional/real-time RT-PCR assays have been apricated for BTV surveillances in mainland China and Korea (137–139, 141, 143–145). The agar gel immunodiffusion (AGID) test for detection of antibodies to BTV also had been used (131). However, cross-reactions between IBAV and BTV may result in false-positive to BTV-AGID (153). There is no available information for applicable BTV vaccines in East Asia.

### Other Orthobunyaviruses

Around the year 2000, the circulation of three orthobunyaviruses (genus *Orthobunyavirus*) which were new in East Asia was identified. In 1999, Peaton virus (PEAV; species *Peaton orthobunyavirus*), which was initially isolated in Australia in 1976 (154), was obtained from sentinel cattle and *Culicoides* biting midges in Japan (155). A stocked virus isolated in 1987 was also identified as PEAV (107). Australian researchers reported that the experimental infection of a pregnant ewe with PEAV induced congenital defects in its lamb (156). Precolostral sera obtained from 31 malformed calves between 1996 and 2016 were positive for PEAV, but negative for known teratogenic viruses (157). These findings suggested that, like AKAV and AINOV, PEAV causes congenital malformations in calves and lambs. Antibodies to PEAV were detected in cattle in mainland China during large-scale sero-surveillance for AKAV (83). The seroprevalence (1.1%) of PEAV among cattle was also demonstrated in Korea during 2013–2015 surveillance (78). In Taiwan, a partial sequence of PEAV was obtained from *Culicoides* biting midges (109). The PEAV circulation was also observed in Israel (158). During the epizootic, PEAV specific gene was detected from a calf with hydranencephaly (159).

Sathuperi virus (SATV; species *Schmallenberg orthobunyavirus*) was isolated from mosquitoes in India in 1957, and its prevalence in Nigeria in the 1960s was identified (160, 161). The virus has been isolated from sentinel cattle and *Culicoides* biting midges since 1999, and an association between SATV and congenital abnormalities in calves was suggested because precolostral sera from several calves with congenital malformations neutralized SATV (162, 163). The seroprevalence (4.9%) of SATV in Korea was also reported (78).

Shamonda virus (SHAV; species *Schmallenberg orthobunyavirus*) was initially isolated in Nigeria in 1960s (161), and since then there had been no report of it for a long while. In 2002, SHAV suddenly emerged in southern Japan, which is geographically far from Africa (164). Precolostral sera from several malformed calves delivered after the SHAV spread in the epizootic region contained neutralization antibodies to SHAV (164–166). Between the winter of 2015 and the spring of 2016, over 20 malformed calves exposed to intrauterine infection with SHAV were reported in southern Japan (166, 167). Antibodies to SHAV (5.6%) were also detected in Korea, but there is no information regarding its association with abnormal deliveries (78).

The pathogenicity of these orthobunyaviruses is poorly understood, because no experimental infections to cattle successfully developed clinical signs that are observed in the clinical cases in the field. The reported cases that were probably affected by PEAV, SATV, or SHAV were highly sporadic after their epizootic in Japan. The congenital lesions in the brains of calves infected with PEAV or SHAV were milder than those of calves infected with AKAV or AINOV (157, 166). It is probable that the viruses currently circulating in East Asia have relatively low pathogenicity. However, like other segmented viruses, a genetic shift of orthobunyaviruses with three segmented genomes potentially strengthens their virulence to ruminant hosts (168).

It is of interest that the “S” and “L” RNA segments of SBV have a high degree of sequence identity with that of SHAV, but the sequence of the “M” RNA segment of SBV was genetically close to that of SATV (163, 169). *In vitro* coinfection assays in mammalian and insect cells easily generate reassortants between SATV and SHAV (170). The genetic reassortment might have also occurred among other orthobunyaviruses isolated from ruminants in East Asia and Australia in their evolutional history (70, 107). Although the impacts of PEAV, SATV, and SHAV remain unclear, these viruses should be listed among the teratogenic viruses in ruminants, and their circulation and the suspected cases associated with them should be monitored in East Asia.

### Orbiviruses Other Than IBAV, CHUV, and BTV

In 1997, over 1,000 cases of abortion and stillbirths in cattle occurred in southern Japan. A potential etiological agent was isolated from aborted fetuses, sentinel cattle, and *Culicoides* biting midges and was identified as a strain of EHDV (65, 171, 172). The EHDV strain was initially regarded as a variant of IBAV, because the virus was cross-reacted with IBAV by a virus neutralization test, and the sequence of its segment 2 was reported to show a degree of similarity to that of IBAV (172, 173). Later research demonstrated that this virus belongs to the serotype 7 group of EHDV, based on phylogenetic studies of segment 2 sequences encoding the outermost capsid protein (46, 174). Ibaraki disease-like symptoms (e.g., swallowing difficulty) in cattle were also reported during the epizootic. In 2013, EHDV-7 was also isolated in the southern part of mainland China from sentinel cattle, but its pathogenicity remains unknown (175).

In 2015, an outbreak of febrile illness resembling Ibaraki disease in cattle occurred in a limited part of mainland Japan (176). The EHDV serotype 6 (EHDV-6) gene was detected in all affected cattle by serotype-specific RT-PCR. Although EHDV serotype 1 has been detected and isolated in Japan (46, 174, 177), its association with clinical cases in ruminants has not been demonstrated. A strain of EHDV that was isolated in the southwestern islands of Japan was serologically and genetically distinct from the known EHDV serotypes and was tentatively assigned to the new serotype 10 (174).

D'Aguilar virus (DAGV), which is a member of the species *Palyam virus*, was first isolated in Australia in 1968 (110). The analysis of stocked viruses revealed that the incursion of DAGV occurred in Japan's southwestern islands in 1987 at the least (178). DAGV spread in southern Japan in the autumn of 2001, and affected calves with Chuzan disease-like symptoms were observed during the following winter and spring. During the epizootic period, no CHUV was isolated from cattle in the affected region. Precolostral sera of these calves clearly neutralized DAGV, suggesting its association with congenital malformations. The virus has been repeatedly isolated in the southern part of mainland Japan and Japan's southwestern Islands (179, 180).

Bunyip Creek virus (BCV), which is another serotype of species *Palyam virus*, was isolated from cattle and *Culicoides* biting midges in Japan in 2008 and 2009 (179, 180). There is no evidence that BCV was associated with ruminant disease in Japan or in Australia, where BCV was initially isolated (181).

Yunnan orbivirus (YUOV) was first isolated from mosquitoes collected in the southern part of mainland China in 1997, and it was identified as a member of a new orbivirus species, *Yunnan orbivirus*, in 2005 (182). A second serotype of *Yunnan orbivirus* (YOUV-2), which is also called Middle Point virus, was obtained from healthy cattle in Australia in 1998 (183, 184). In addition, another orbivirus related with YOUV was isolated from a blood sample of cattle in the southern part of mainland China in 2015 and was designated as Guangxi orbivirus (185). Interestingly, YOUV was also isolated from cattle, sheep, a donkey, and a dog in Peru in 1997 (186). The infected animals showed neurological signs. Although no evidence of an association between YOUV and animal disease in East Asia has been described, this finding suggests its potential ability to cause neurological disease in domestic ruminants.

## Zoonotic Arboviruses

### Mosquito-Borne Arboviruses

Several zoonotic arboviruses of importance to human health have been detected from domestic ruminants in East Asia (Table 4). Some of these arboviruses cause clinical signs in both infected humans and ruminants, but the others produce asymptomatic and negligible mild disease in ruminants.

**Table 4:** Zoonotic arboviruses infecting domestic ruminants in East Asia.

**Virus**	**Symptoms in humans**	**Symptoms in domestic ruminants (species)**
BATV	Mild flu-like illness	Mild illness (sheep, goat)
SFTSV	Severe fever with thrombocytopenia syndrome	NR
CCHFV	Hemorrhagic fever	NR
BAV	Encephalitis	NR
JEV	Encephalomyelitis	Encephalomyelitis (cattle)
WNV	Febrile illness, encephalitis, meningitis	Lymphoplasmacytic meningoencephalitis (sheep)
KFDV	Hemorrhagic fever	NR
TBEV	Febrile illness, encephalitis	NR

*NR, none reported*.

Japanese encephalitis virus (JEV; species *Japanese encephalitis virus*, genus *Flavivirus*, family *Flaviviridae*) caused severe encephalitis not only in humans but also in horses and cattle (187). The JEV infection resulted in abortion and stillbirth in sows and aspermia and disruptions of spermatogenesis in boars (187). Although JEV has spread across a wide area in Japan (188), only a few affected cases of cattle have been sporadically recorded in Japan (189–193).

Outbreaks of West Nile virus (WNV; species *West Nile virus*, genus *Flavivirus*) infection have occurred across the Mediterranean region, Eastern Europe, Africa, Asia, and North America (194). Human cases of WNV infection were also reported in western China in 2011, and WNV was simultaneously isolated from mosquitoes in the affected area (195). The WNV gene was detected from domestic pigeons in Korea in 2014 (196). It was recently demonstrated that sporadic cases of severe lymphoplasmacytic meningoencephalitis were associated with WNV in California (197). However, ruminants are not considered hosts severely affected by WNV, and no ruminant illness associated with WNV has been recorded in East Asia.

Banna virus (BAV), which belongs to the species *Banna virus* of the genus *Seadornavirus* within the family *Reoviridae*, was first isolated in mainland China in 1987 from a patient with meningoencephalitis (198, 199). Since then, BAV has been obtained from pigs, cattle, ticks, mosquitoes, and *Culicoides* biting midges over a wide range of mainland China (200, 201). The virus is thought to cause encephalitis in humans, and many cases associated with it might have been misdiagnosed as Japanese encephalitis (200). At present, there is no evidence of a relationship between BAV and ruminant illness.

Batai virus (BATV), which belongs to the species *Batai orthobunyavirus* of the genus *Orthobunyavirus*, has been isolated in Japan and mainland China since 1994 (202–205). BATV is widely distributed in Asia and Europe, and its infection has been observed in cattle, sheep, and goats (202, 206, 207). BATV was reported to cause a mild flu-like illness in humans and mild illness among sheep and goats. At present, no affected case associated with BATV has been reported in East Asia. Ngari virus, which is a possible reassortant between Bunyamwera virus and BATV, has been implicated in human hemorrhagic fever in Africa (202, 208, 209). In the future, BATV may obtain a higher virulence by genetic reassortment with other related orthobunyaviruses. The potential risk of BATV to public health in East Asia should be considered.

### Tick-Borne Arboviruses

Four medically important tick-borne arboviruses that infect domestic ruminants are prevalent in East Asia (Table 4). Crimean-Congo hemorrhagic fever virus (CCHFV; species *Crimean-Congo hemorrhagic fever orthonairovirus*, genus *Orthonairovirus*, family *Nairoviridae*) was involved in human clinical cases in the northwestern part of mainland China (210). Although infected domestic ruminants play a crucial role as reservoirs of CCHFV in an endemic cycle of transmission, they do not develop any symptomatic illness after CCHFV infection (211).

Tick-borne encephalitis virus (TBEV; species *Tick-borne encephalitis virus*, genus *Flavivirus*, family *Flaviviridae*) is widely distributed in mainland China, Japan, and Korea (212). This virus sometimes causes severe encephalitis in humans. In Europe, TBEV infection in humans following the consumption of unpasteurized milk and cheese from domestic ruminants has been reported (213). However, the role of domestic ruminants in TBEV endemics in East Asia remains unknown. Another zoonotic tick-borne flavivirus, Kyasanur Forest disease virus (KFDV), was isolated from a febrile patient in the southern part of mainland China in 1989 (214). Although domestic ruminants may be infected with KFDV (215), their role in the viral transmission remains unclear.

Severe fever with thrombocytopenia syndrome virus (SFTSV; species *Huaiyangshan banyangvirus*, genus *Banyangvirus*, family *Phenuiviridae*) was recognized in mainland China, Japan, and Korea (216–218). Although this tick-borne virus has probably been circulating in East Asia for many years, its association with the severe illness in humans was not recognized until 2011. The seroprevalence of SFTSV in domestic ruminants in mainland China, Japan, and Korea was reported (219–222), and epidemiological studies suggested that farmers in rural areas are at risk of SFTSV infection (223). The continuous monitoring for SFTSV in domestic ruminants is therefore necessary to assess the ruminants' contribution to SFTSV endemics in the affected regions.

Compared to mosquito/*Culicoides*-borne viruses, tick-borne viruses are not likely to expand their range within a short period of time. However, it was suggested that migratory birds play an important role as long-distance transporters for ticks infected with arboviruses (224, 225). The role of birds in any sudden incursion and occurrence of tick-borne virus infections in a region that was previously free of the infections should thus be considered.

## Other Arboviruses

Several arboviruses with unknown impacts have been confirmed to infect domestic ruminants. The infected ruminants usually do not develop apparent clinical signs, and thus these viruses have been neglected for many years. Nevertheless, the recent progress in genetic analyses can characterize their properties and assign their taxonomy.

Getah virus (GETV; species *Getah virus*, genus *Alphavirus*, family *Togaviridae*) is an etiological agent of fever, rash, and leg edema in horses (226) and of fetal death and reproduction disorders in pigs (227, 228). The prevalence of GETV has been confirmed in mainland China, Japan, Korea, and Taiwan (229, 230). Outbreaks of GETV infection have occurred among Japanese racehorses (231, 232). It is known that GETV also infects cattle, although there is no evidence of illness in the infected cattle (226, 233, 234). However, cattle may contribute to a GETV endemic as a reservoir.

Fukuoka virus (FUKV) is assigned to the species *Fukuoka ledantevirus* of the genus *Ledantevirus* within the family *Rhabdoviridae* and was initially isolated from *Culicoides punctatus* and the mosquito *Culex tritaeniorhynchus* in southern Japan in 1982 (235). The virus was first regarded as a member of the ephemeral fever serogroup but was subsequently classified as a member of the Kern Canyon serogroup viruses (236, 237). FUKV was later isolated from sentinel cattle in a different region in Japan and, simultaneously, the seroprevalence of FUKV in cattle was demonstrated (238). Although cattle were identified as a susceptible host of FUKV, the pathogenicity of this virus in cattle remains uncertain. Full-genome analysis of ledanteviruses demonstrated that FUKV has a closed relationship (79.5% nucleotide identity) with Barur virus which has been isolated from ticks, fleas, mosquitoes and a rodent in India and Africa (239). Nishimuro virus from a wild boar in Japan (240) is also genetically similar to FUKV. Further studies are needed to determine their vertebrate hosts and arthropod vectors.

Sathuvachari virus (SVIV) is a member of the genus *Orbivirus* and was initially isolated in 1963 from starlings (*Brahminy myna*) collected in southern India (241). The virus was also isolated from an unidentified source collected in Vietnam over 50 years ago (242). The genetic characterization of SVIV suggested its close relationship with other mosquito-borne orbiviruses (241). An unassigned virus isolated from bovine blood collected in Japan's southwestern islands in 2005 was recently identified as SVIV (243). No illness in the infected cattle was reported when their blood samples were collected, but the finding demonstrated that cattle are a susceptible host of SVIV and indicated that the virus has a wider host range than previously thought.

An ephemerovirus tentatively called KSB-1/P/03 was isolated from sentinel cattle in southern Japan in 2003 (179). The phylogenetic analysis based on the L protein of rhabdoviruses revealed that this virus is clustered with other ephemeroviruses. The infected cattle did not demonstrate any clinical signs. Other than BEFV, no ephemerovirus had been isolated in East Asia. Further genetic analyses should be conducted to determine the taxonomic assignment of KSB-1/P/03.

A virus isolated from mosquitoes in Tibet, China in 2009 was identified as a novel species of the genus *Orbivirus* (244). Designated as *Tibet Orbivirus* (TIBOV), the virus has been obtained from mosquitoes and *Culicoides* biting midges in other regions of mainland China (86, 245, 246). Phylogenetic analysis of inner core (T2) protein of known orbiviruses demonstrated that TIBOV is clearly categorized into the *Culicoides*-borne group with BTV, EHDV, and PALV (244, 245). The amino acid sequence diversity among outermost capsid proteins of TIBOV isolates indicated that the species consists of at least two serotypes (246). The neutralization antibodies to TIBOV were detected in cattle reared in the southern border region of mainland China (246).

A novel segmented RNA virus which is genetically related to flaviviruses was detected in ticks collected in mainland China in 2010 and was designated as Jingmen tick virus (JMTV) (247). Together with insect viruses that have a similar genome composition, JMTV is tentatively referred as the Jingmenvirus group (248). Cattle sera collected in mainland China were reported to contain antibodies against JMTV (247). The genome of JMTV was also detected in a part of the seropositive samples by RT-PCR screening. JMTV was recently identified as a potential human pathogen, because patients infected with the virus showed clinical manifestations such as fever, headache, and myalgia (249).

## Arthropod Vectors of Important Ruminant Arboviruses

The identification of the vectors of arboviruses is essential to understand the epidemiology of the arboviruses and to develop control measures against them. In this section, we focus on the potential vectors of arboviruses that affect domestic ruminants in East Asia. Many orbiviruses (e.g., BTV and EHDV) and orthobunyaviruses (e.g., AKAV and SBV) that are pathogenic to domestic ruminants were reported to be transmitted by *Culicoides* biting midges (250, 251). Long-term monitoring for virus detection in *Culicoides* biting midges in southern Japan demonstrated that various arboviruses such as AKAV, AINOV, SATV, CHUV, DAGV, BCV, and EHDV were preferentially isolated from *C. oxystoma* (Table 5) (65, 179).

**Table 5:** Isolation sources of hematophagous invertebrates for arboviruses affecting domestic ruminants in East Asia.

**Virus**	**Mosquitoes**	***Culicoides* biting midges**
AKAV	*Aedes vexans, Culex tritaeniorhynchus, Cx. quinquefasciatus, Anopheles sinensis, An. vagus*	*C. oxystoma*
AINOV	*Cx. tritaeniorhynchus*	*C. oxystoma, C. punctatus*
PEAV	NR	*C. jacobsoni*
SHAV	NR	*C. tainanus*
SATV	NR	*C. oxystoma*
CHUV	NR	*C. oxystoma*
DAGV	NR	*C. oxystoma, C. sumatrae*
BCV	NR	*C. oxystoma*
EHDV-1	NR	*C. punctatus*
EHDV-2	NR	*C. oxystoma, C. lungchiensis, C. punctatus*
EHDV-7	NR	*C. oxystoma*
BTV-2	NR	*Culicoides* spp.
BTV-9	NR	*C. asiana*
BTV-12	NR	*Culicoides* spp.
BTV-16	NR	*C. tainanus*
YUOV	*Cx. tritaeniorhynchus*	NR

*NR, none reported*.

Although *Culicoides* biting midges are regarded as incriminate vectors of AKAV, the virus has been obtained from three major mosquito genera (*Aedes, Culex*, and *Anopheles*) in mainland China and Japan (Table 5) (60, 84, 86). A small portion of mosquitoes that feed on infected cattle during viremia may be infected with AKAV, as observed in the experimental infection of *Culex* mosquitoes with SBV (252).

*Culicoides* species such as *C. imicola* and *C. sonorensis*, which play the principal role for ruminant arbovirus transmission in other regions, are not distributed in East Asia (253). The distribution of *C. brevitarsis*, which is a incriminate vector of ruminant arboviruses in Australia (254) was described in southern Japan and Taiwan (255–257). However, molecular analyses using mitochondrial and nuclear DNA indicated that the specimens collected in southern Japan were distinct from the *C. brevitarsis* specimens collected in Australia, and thus a new species was recorded: *Culicoides asiana* (258, 259). The isolation of BTV from field-collected *C. asiana* was successful (260), and an experimental infection indicated that this species is susceptible to AKAV (261).

These findings suggested the vector capacity of *C. asiana* as with *C. brevitarsis*. The above-cited studies also suggested that *C. brevitarsis* and *C. asiana* were co-distributed throughout Southeast Asia and its neighboring regions, including Hainan Island, which is in a subtropical zone in China (258, 259). The role of these two species in arbovirus transmission should be further evaluated in their distribution ranges.

A laboratory rearing system of *Culicoides* biting midges was successfully established for limited species distributed in North America and Europe (251, 253). This limitation hampers the progress in the assessments of the vector capacity of various other *Culicoides* species. Instead, field-collected midges have often been used for checking the oral susceptibility and vector competence of ruminant arboviruses (262–266). Unfortunately, the vector competence of *Culicoides* biting midges distributed in East Asia is poorly understood. The viral isolations from pools of field-collected midges which were identified at the species level have helped estimate vector species (65, 179).

The oral susceptibility of several Japanese *Culicoides* species for AKAV was demonstrated (261). As the virus isolation from field-collected midges suggested, *C. oxystoma* was susceptible to AKAV, and the viral replication and dissemination were demonstrated in the infected midges. Interestingly, *C. tainanus* and *C. punctatus*, which have not been considered AKAV-competent vectors, were also orally infected with AKAV. During the AKAV endemic in northern Japan in 2010, *C. tainanus* and *C. punctatus* were dominant/subdominant species at cowsheds in the endemic region, but *C. oxysoma* has been never collected there (267). These species, which are common in East Asia (268–271) probably contributed to the AKAV transmission during that period.

BEFV has been isolated from several mosquito and *Culicoides* species in Africa, Australia, and Central Asia (9), but there has been no record of the isolation of BEFV from invertebrate vectors in East Asia so far (28). A laboratory experimental infection of *Culicoides* biting midges indicated that, at the least, they are not efficient vectors of BEFV (272). The vector search will be essential to understand the epidemiology of BEFV in East Asia.

In light of the BTV and SBV endemic experiences in northern Europe, it appears that the *Culicoides* species that are indigenous in northern temperate regions have the potential to transmit ruminant arboviruses (273, 274). It is necessary to assess the vector capacity of *Culicoides* species which are distributed in the higher-latitude regions in East Asia. It is also important to determine their distribution range and seasonality to gain a greater understanding of the potential risks of arbovirus spreads, even in previous arbovirus-free regions.

## Research Gaps and Future Directions

Vaccinations are probably the most effective preventive measures for arbovirus infections in domestic ruminants. The numbers of animals affected by AKAV, AINOV, CHUV, IBAV, and BEFV were dramatically reduced in Japan after the spread of vaccinations (52). However, limited effectiveness of the current vaccination strategy for BEFV was suggested in Taiwan and Israel (33, 275). It might be difficult to confer long-lasting effective immunity in vaccinated and infected cattle (276, 277), and this probably caused the repetition of BEF occurrence in the above-mentioned regions. It remains difficult to prevent the encephalomyelitis caused by postnatal AKAV infection (67). Vaccines for AKAV are generally administered to breeding cows but not other cattle such as fattening cattle and bulls. There is not yet enough preparation for the pathogens that have recently emerged, such as PEAV, SATV, SHAV, and YUOV, which are suspected to cause ruminant illnesses. Probable loss and future threat caused by these viruses also remain uncertain. Depending on the situation, the development of preventive measures for these arboviruses that have newly emerged in East Asia would be necessary.

The genogroup shifts of AKAV in Japan clearly reflected the change in the types of disease (67). The newly emerged clade of BEFV seems to be more virulent than those of previously endemic strains (28). In addition, the reassortment of segmented RNA viruses (such as orthobunyaviruses and orbiviruses) may increase the viral fitness in vertebrate hosts and/or invertebrate vectors and enhance the virulence in vertebrate hosts and/or the transmission efficacy by invertebrate vectors. Therefore, continuous monitoring of the virulence and pathogenicity of endemic arboviruses should be conducted in East Asia.

The recent emergence of “new” arboviruses in East Asia has attracted interest in their origins and incursion routes. In addition, genogroup shifts of endemic arboviruses in the region have probably been induced by the introductions of viruses from a different genetic pool in other regions. Extensive surveillance in Australia obtained many arboviruses, and some of them are also endemic or were recently emerged in East Asia (278). However, phylogenetic studies of orthobunyaviruses and BEFV indicated that they evolved independently in separate gene pools with different geographical origins (22, 108, 279). In contrast, BEFV, BTV, BANV, and JEV strains, which were isolated in Southeast Asia, often have closed genetic relationships with those isolated in East Asia (280–283).

The circulation of other ruminant arboviruses such as AKAV, AINOV, PEAV, and BATV has also been demonstrated in Southeast Asian countries by serological surveillance and virus isolations (284–287). Unfortunately, there are not enough reports to determine the current status of these arboviruses in Southeast Asia. Further virological surveillance and genetic characterizations of isolated viruses are essential to elucidate the epidemiological linkage of ruminant arboviruses between East Asia and Southeast Asia.

The long-range dispersal of vector insects by winds is considered a principal cause of the introduction of arboviruses to geographically distant locations, including previously arbovirus-free areas (288). Historically, windborne introductions of *Culicoides*-borne animal diseases from distant endemic areas have been estimated based on meteorological data (289, 290). Plausible dispersal events from eastern Indonesia, Timor-Leste, and southern Papua New Guinea explored by using an atmospheric dispersion model helped elucidate the incursion of exotic *Culicoides* species and BTVs into northern Australia (291). Atmospheric dispersion models would be useful to assess the potential sources of arbovirus incursions from outside of East Asia and their spread within the region.

As a pioneering study, a backward-trajectory analysis of the BEF epidemic in 2012 in Japan's southwestern islands showed that the estimated potential sources of infected vectors were Southeast Asian countries such as Vietnam and Philippine (21). Reassessments of historical outbreaks of ruminant arbovirus infections with an atmospheric dispersal model could also help determine their incursion sources and spread patterns.

With the exception of BEFV, which has a higher incident rate in cattle (9), almost all of the arboviruses generally result in a low morbidity, asymptomatic/mild illnesses or no apparent acute phase in domestic ruminants, and thus they silently circulate in the endemic regions. Active surveillance such as sentinel and vector surveillance systems are therefore needed for the efficient detection of the arboviruses' circulation in the field prior to disease outbreaks (292). The recent increase in the number of emerging arboviruses in East Asia indicates that other undetected arboviruses may have circulated. The expansion of global transportation may also enhance the risk of arbovirus introductions from geographically distant areas. Global warming will certainly affect the distribution and active period of vectors, and thus the ranges of virus spread will expand to higher-latitude regions.

Although sensitive detection systems are essential to identify the incursion and circulation of arboviruses, the current serological and genetic techniques cannot cover all arboviruses, especially if they are novel. Next-generation sequencing (NGS) technologies will support the detection and characterization of such viruses (293). The findings revealed by NGS will contribute to the development of diagnostic/detection systems suitable for the arboviruses and to the progress of etiological studies.

Clearly, knowledge gaps remain regarding the transmission cycles of arboviruses and vector ecology in East Asia. To the best of our knowledge, there is no detailed distribution map of vectors in the East Asia region. The vector capacity for arboviruses of veterinary importance has been evaluated for only a limited number of species. Further studies of the systematics, biology, and vector competence of mosquitoes, *Culicoides* biting midges, and ticks should be conducted. The knowledge obtained from vector studies will contribute to increased accuracy of epidemiological analyses based on landscape and meteorological data. Risk assessments based on such analyses will enable the development of better surveillance and preventive measures for arbovirus infections which threaten the production of ruminant livestock, as suggested in earlier investigations (294, 295).

Finally, the construction of a network in East Asia and its neighboring countries would be essential to promote research on arbovirus infections of importance to livestock. The sharing of epidemiological information, diagnostic tools, and control/preventive strategies are highly important because neighboring countries are likely to experience the same problems due to arboviruses. Language barriers may present a challenge to information exchanging and sharing. Many scientific reports and public records from East Asian countries have been written in national languages such as Chinese, Japanese, and Korean, but a limited portion of them might have been translated in English. Unfortunately, the status of arbovirus infections in domestic ruminants in Mongolia and North Korea remains unclear. Regional cooperation would therefore be a crucial key to clarify the current status of arbovirus infections and minimize their impacts on the livestock industries in East Asia.

## Author Contributions

TY executed a first draft. YH and KM helped draft the manuscript. All authors contributed equally to the final draft and editing of the manuscript.

### Conflict of Interest

The authors declare that the research was conducted in the absence of any commercial or financial relationships that could be construed as a potential conflict of interest.
